# Interventional cardiovascular magnetic resonance: still tantalizing

**DOI:** 10.1186/1532-429X-10-62

**Published:** 2008-12-29

**Authors:** Kanishka Ratnayaka, Anthony Z Faranesh, Michael A Guttman, Ozgur Kocaturk, Christina E Saikus, Robert J Lederman

**Affiliations:** 1 Translational Medicine Branch, Division of Intramural Research, National Heart Lung and Blood Institute, National Institutes of Health, Bethesda, Maryland, USA; 2 Cardiology Division, Children's National Medical Center, Washington, DC, USA

## Abstract

The often touted advantages of MR guidance remain largely unrealized for cardiovascular interventional procedures in patients. Many procedures have been simulated in animal models. We argue these opportunities for clinical interventional MR will be met in the near future. This paper reviews technical and clinical considerations and offers advice on how to implement a clinical-grade interventional cardiovascular MR (iCMR) laboratory. We caution that this reflects our personal view of the "state of the art."

## Background

### Why interventional CMR?

Surgical procedures afford direct *exposure *for visualization (optical imaging) of target tissue and *access *for mechanical intervention. This permits instant recognition of important complications like bleeding to allow immediate correction during the procedure. Unfortunately surgical exposure is morbid, meaning it produces physiological and geometric derangement, discomfort, immobilization, and risk.

Catheter-based (*i.e*. endovascular) approaches have revolutionized the treatment of cardiovascular disease. X-ray fluoroscopy guides a wide range of minimally invasive procedures, including treatment of obstructive coronary artery disease, peripheral artery atherosclerosis and aneurysm, and structural or congenital heart disease. X-ray infrastructure is widely deployed, and versatile catheter devices are commercially available. X-ray fluoroscopy depicts soft tissue structures poorly and requires skilled operators to make assumptions about anatomic landmarks that may be inaccurate. X-ray exposes patient and operator to ionizing radiation that increases with procedure complexity, and significant career musculoskeletal injury to operators forced to wear protective lead aprons.

While the actual risk of radiation injury remains controversial, even low-level exposure to ionizing radiation is thought to contribute to the long-term risk of malignancy [1,2]. Children are considered more sensitive to radiation and may live longer to experience radiation toxicity. Children requiring catheterization for congenital heart disease often require multiple procedures over time. Chromosomal damage was evident in the peripheral blood of children exposed to catheterization-related radiation [3].

The great promise of iCMR is to provide some of the soft-tissue visualization afforded by direct surgical exposure with the reduced morbidity of X-ray catheter procedures. MR is attractive in the ability to display different blood and tissue contrasts, motion, flow, *etc *without ionizing radiation. This comes at the expense of increased operational complexity, reduced temporal and spatial resolution, and limited availability of catheter devices that are safe and visible under MR. For certain applications, these difficulties might be justified. Complex and prolonged procedures in children who are especially sensitive to long-term toxicity from radiation exposure, is one example. New interventional alternatives to conventional open surgery are another. Improved recognition and response to iatrogenic injury, such as perforation, is yet another.

### Real-time interactive MR

Diagnostic MR utilizes longer image acquisition (for example ECG-gated segmented MR) and off-line reconstruction for higher image quality. Real-time MR combines acquisition, reconstruction, and display to provide operators images with low latency, empirically less than 250 ms compared with 100 ms for X-ray fluoroscopy. X-ray fluoroscopy produces 15 relatively sparse frames per second with a pixel matrix of 1024 × 1024 and a field of view of 8–40 cm. In real-time MR, the imaging matrix (and thus spatial resolution) is reduced in size to support an imaging speed between 5 and 10 frames per second [4]. Even at a reduced pixel imaging matrix such as 192 × 128 at 8 acquired frames per second, real-time MR is comparably information-rich. Most modern real-time MR implementations employ balanced steady state free precession (SSFP, also known as *TrueFISP, bFFE, or FIESTA*) techniques because of efficient use of magnetization, high SNR, and short repetition times. Fast spin echo techniques produce excellent image quality, but are less attractive for real-time applications because of higher specific absorption rates (SAR) of heat from radiofrequency energy and longer acquisition times. In addition to conventional rectilinear sampling strategies, spiral and radial trajectories are also well-suited for real-time imaging [5,6].

Image acquisition speeds may be accelerated through partial and/or parallel acquisition schemes. Partial acquisitions asymmetrically skip some data acquisition in either the frequency (partial echo) or phase encoding direction, and the missing data is synthesized with techniques such as homodyne reconstruction [7] or projection onto convex sets [8]. Parallel acquisition schemes acquire undersampled datasets by skipping some phase-encoding lines, and exploit the sensitivity profiles of multiple coils to reconstruct the missing data. The two most commonly used algorithms for parallel image reconstruction are SENSE [9,10] and GRAPPA [11]; with specialized coil arrays and sufficient computational horsepower, four-fold acceleration rates are typically achieved [12] (*vide infra*). The computational demands on image reconstruction are greatly increased, but real-time interactive imaging with TSENSE has been demonstrated using computers with multiple CPUs [13].

The major commercial MR systems provide real-time SSFP capability with interactive graphic slice prescription during continuous scanning. Some interventional implementations use separate external reconstruction computer systems operating in parallel, having bidirectional links for scan control, providing rapid image reconstruction. Guttman, McVeigh, and Ozturk developed such a comprehensive interactive real-time MR reconstruction system for interventional applications at NIH [14]. This system features real-time imaging of multiple slices and displays these slices in their relative three-dimensional position. Perhaps most important is the ability independently to process and highlight signals from individual receiver channels, especially those attached to intravascular devices [15]. Highlighted devices are then easily distinguished from each other and surrounding tissue, providing high user confidence in iCMR guidance. Other interactive features we have found useful include device-only projection imaging, electrocardiographic gating to suspend cardiac motion and saturation pre-pulses to enhance the appearance of gadolinium during injections into arteries, devices, or myocardium having late gadolinium enhancement. Image-acceleration is controlled interactively. It has proven useful to apply subtraction masks for interactive real-time digital-subtraction MR angiography [16].

Santos and colleagues at Stanford University have developed a real-time MR system (*RTHawk*), which allows dynamic switching between pulse sequences and reconstruction algorithms [17]. This system enables the user to rapidly switch between low spatial resolution localizers and high spatial resolution images, analogous to "fluoroscopy" and "cineangiography" X-ray modes. The system also accommodates active device tracking by interspersing 3 orthogonal projections for device localization and real-time overlay of the device position on anatomical images.

Device-scanner interaction also can be automated. For example, the group at Case Western Reserve University automatically increases temporal resolution and field of view when rapid device motion is detected, then increases spatial resolution and decreases field of view when device motion is low [18]. The effect is to "zoom in" and slow down for more accurate imaging during fine device positioning [18].

Our investigational interventional procedures are guided by multiple slices acquired and displayed in rapid succession. A three-dimensional volume-rendering of the slices conveys their relative positions. Updates of individual slices can be paused or reactivated as needed during a procedure. This type of display can further be enhanced by permitting the user interactively to apply point markers on important anatomic features or targets, make measurements on-line, and combine with prior roadmap images to make rapid before-after comparisons.

Many of these concepts have been incorporated into an "Interactive Front End" system developed by Christine Lorenz of Siemens Medical Systems and available for investigational use [19].

**Figure 1:**
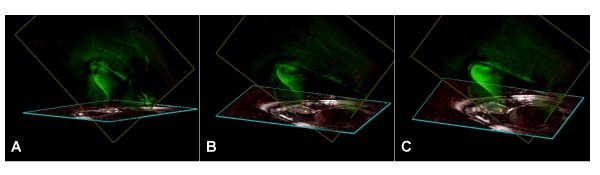
**This is an example of "adaptive" projection navigation**. Two dimensional slices are volume-rendered to show their three-dimensional position. In addition, the active catheter device is shown in superimposed projection-mode. The viewing angle of the two-dimensional slices can interactively be changed [A-C], but in this mode the projection angle is always perpendicular to the selected viewing angle. In this example, an active myocardial injection catheter is navigated toward an infarct border. The catheter contains two receiver coils: a small coil at the tip (red) and a loopless antenna along the shaft (green). A yellow dot is applied by the operator to mark the tissue target. The volume rendering is rotated manually during the procedure to view the device trajectory from different angles, conveying three-dimensional information [14]. In A and B, the catheter tip is outside the imaging plane; in C the catheter tip is in-plane and coincides with the selected target.

iCMR operators have two general imaging approaches. For some applications, imaging is directed at target pathology, while devices are manipulated in or out of the desired target slice. Alternatively, the real-time imaging slice can automatically track catheter movements and alter the slice prescription so as always to keep the desired catheter device in view, while changing the view of the neighboring anatomy. Ideally, both imaging techniques should be available to the operator. We have found a "projection-mode" feature useful during catheter manipulations within target slices. When parts of catheter devices move outside of a typical thin-slice image, they are no longer visible to the operator. By toggling projection-mode (which switches to thick-slice or non slice-selective imaging), the catheter can be "found" and manipulated back into the target slice. Combining an "adaptive" projection mode (automatically orthogonal to the selected viewing angle) with multi-slice three-dimensional rendering has been especially useful (Figure 1).

### Outfitting a laboratory to perform interventional CMR procedures

This section attempts to answer questions raised during site visits. Readers are cautioned our recent first-hand experience is limited to one system vendor.

#### How do we site the imaging equipment? How do we justify an interventional MR system when there are no remunerative iCMR procedures?

**Figure 2:**
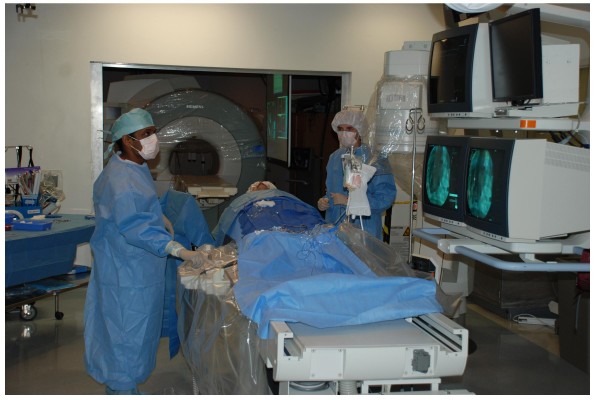
**A combined X-ray and MRI interventional suite**. The two systems can be used independently or together, when the radiofrequency-shielded and leaded doors are retracted. Patients can be transferred between labs on a cradle that slides along a motorized table. Each room can display real-time images, instantaneous hemodynamics, and imaging control to the operator. Both share a common control room for support staff [45].

A lab for investigational interventional procedures should be configured with emergency bailout procedures in mind. We recommend interventional CMR laboratories adjoin conventional X-ray systems. Adjoining X-ray and MR systems can be configured to operate independently or together. The only incremental capital outlay required is for an intermodality patient transport system and for doors that separate the adjoining labs. Intermodality transport systems are available from all major system vendors and generally consist of floor-mounted rails or free-moving trolleys conveying a multimodality patient cradle or shell. Doors can maintain the radiofrequency shield of the MR lab and can contain lead if separating distance doesn't provide enough radiation protection. Doors can be motorized or manually-operated. One vendor (IMRIS) mounts an MR system on ceiling tracks for movement between multiple operating rooms or interventional suites while the patient table remains stationary. A common control room for the adjoining MR and X-ray can reduce additional costs. Video systems can substitute for radiofrequency-shielded windows into the MR lab (Figure 2).

Conversely, co-localizing X-ray and MR systems within a single radiofrequency shield does not appear a cost-effective solution, because the systems cannot be used independently. Indeed X-ray systems generally need to power-down because they generate radiofrequency noise during MR. The "cold" start-up time for X-ray systems can be onerous if emergency bailout imaging is required. Mobile C-arm X-ray systems generally provide inferior imaging and gantry positioning and are unattractive for high-risk cardiovascular interventional procedures.

All intermodality transfers should be smooth, rapid, and convenient. Configurations that require complex rotation of the patient table in order to move between X-ray and MR are none of these. Indeed, patients often are attached to bulky appliances such as pumps and ventilators that might also need to travel as the patient table is rotated. For dual-use suites, siting plans should provide separate patient entry and egress for the X-ray system that is outside the high (5 gauss) magnetic field, especially when many patients undergoing conventional catheterization may have "MR unsafe" or "MR conditional" cardiovascular implants [20].

Interventional procedures have been conducted in MR systems ranging from low-field (<1.0 T) open magnets to long bore high-field (3.0 T) systems. While open systems allow easier patient access, they tend to have inferior image quality. High-performance SSFP is available in 1.5 T systems and becomes more challenging at higher fields because of inhomogeneity of the main field and higher SAR. Shorter- and wider-bore systems are available at 1.5 T and 3.0 T that enhance operator access to the patient, but require compromises in main field homogeneity and head-to-foot field of view.

#### Hemodynamic monitoring and recording

During interventional procedures patients receive medications that may depress spontaneous breathing, or they may suffer sudden hemodynamic deterioration. These procedures therefore require a higher level of human and technical vigilance than does diagnostic MR. Commercially available MR hemodynamic monitoring solutions may not be adequate.

Comfort and respiratory effort are best monitored by direct visualization. If this is not feasible, video and lighting equipment should be installed inside and outside the magnet bore to permit the staff to assess patient respiratory efforts, comfort, and even skin color as an index of perfusion or autonomic state.

Hemodynamic "monitoring" systems are distinguished from "recording" systems. The former is for keeping patients safe, the latter for obtaining high-fidelity measurements suitable for cardiovascular diagnosis and treatment. Monitoring systems typically have slower sampling rates, fewer available channels, and are considered unsuitable for heart catheterization. Commercially available monitoring systems often can output analog signals for computerized display, albeit with low-fidelity. There are no commercially available clinical hemodynamic recording systems for MR, although non-clinical systems are readily adapted (*e.g. Powerlab*, ADInstruments Inc) for investigational use. We use the same disposable pressure transducers for both X-ray and MR. We believe interventional labs should have the capability to monitor and record at least two instantaneous blood pressure channels (for gradient determination) as well as one intermittent non-invasive blood pressure recording device.

During conventional X-ray procedures, electrocardiographic (ECG) signals are monitored or recorded from four surface electrodes on the limbs and between one and six on the chest. These are connected to long conductive transmission lines that are prone to heating and therefore unsuitable for use in MR. Clinical MR safe ECG electrode leads are typically short, shielded, fewer, and often wireless. Even if MR cardiac gating is not planned, the skin surface should be prepared for good contact with ECG electrodes for cardiac monitoring. Most labs use two sets of ECG electrode leads, one for use in MR and a neighboring set for use in X-ray. Dual-use ECG transmission lines are not commercially available. Laboratories should plan for the inconvenience and hazard of disconnecting and reconnecting ECG leads before each intermodality patient transfer, until a commercial solution is available. Conventional X-ray electrodes must be removed prior to MR.

Electrocardiographic waveforms are altered when the patient is in the magnet and further during scanning. Magnetic gradients introduce ECG noise that can be filtered by commercial equipment. Electrocardiographic repolarization patterns (the "ST-segment"), which under normal conditions can be monitored for evidence of myocardial ischemia or injury, are irrevocably distorted by the magneto-hydrodynamic effect of cardiac and aortic flow of blood ions. Any procedure that might inadvertently injure myocardium or induce direct or paradoxical coronary embolization of atheromata, thrombi, or air should be conducted only with a monitoring plan that includes a surrogate for ECG repolarization. This might include intermittent real-time surveillance of myocardial motion, instantaneous blood pressure monitoring, or more sophisticated approaches.

Numerous cables and devices are connected to patients during interventional procedures (see below, Inputs and outputs for patient monitoring and care during interventional CMR procedures). These should be positioned to permit rapid entry and exit from the magnet bore toward X-ray bailout. Our preference is that cables, tubes, and interventional devices only enter the magnet bore from the side closest to the X-ray. We also believe the operator must have direct access to (in order to secure) intravascular devices throughout intermodality transfer. For transfemoral procedures this means the X-ray table is positioned head-to-foot with regard to the MR, and the patient enters the magnet bore head-first. Operators should verify that conductive cables are kept away from the wall of the magnet bore and are not coiled, since these predispose to heating during MR.

#### Inputs and outputs for patient monitoring and care during interventional CMR procedures

• Line-of-site for visual assessment of breathing and comfort, or video surrogate

• Electrocardiographic electrodes, leads/cables for X-ray (and for MR, if different)

• Invasive blood pressure transducers, at least two channels

• Noninvasive blood pressure cuff (oscillometric sphygmomanometer)

• Hemoglobin saturation ("pulse oximetry")

• Intravenous lines and MR-compatible intravenous pumps, configured to allow free movement of patient between MR bore and X-ray system

• Accessible port for manual infusion of additional medications

• Audio communications system

• Video entertainment system, if implemented

• Patient-controlled alarm

• Surface coils, transmission lines, and connectors

• Interventional coil transmission lines and connectors

• Clips and bags to assure sterility

• Interventional catheterization manifold, typically including a blood pressure transducer, multiple stopcocks, a controlled volume syringe, saline flush, contrast flush, and waste receptacle

• Oxygen tubing, or anesthesia tubing and anesthesia gas monitor

• Temperature sensor

iCMR laboratories should establish a system for safe resuscitation and defibrillation. Defibrillation equipment is not MR safe and can become a projectile inside the high magnetic field. Moreover, defibrillation currents inside the magnetic field generate enough force to knock over the operator. In our laboratories, the X-ray lab serves as the evacuation destination for cardiovascular emergencies.

For video display, LCD projectors are a cost-effective solution. Commercial projectors function well even when incorporated in radiofrequency shields including copper-screens across the lens. Some commercial LCD projectors function well without modification or shielding. We prefer rear-screen projectors, which we find to perform well if they are greater than 2000 ANSI lumens. Unshielded LCD projectors also could transmit through properly situated waveguides. In addition, shielded and boom-mounted LCD displays are commercially available.

Shielded keyboard and mouse solutions are available from commercial vendors, but ordinary USB or Bluetooth keyboards and mice suffice if filtered through a penetration panel. Some laboratories also use foot-pedal controls to start and stop MR scanning, or to manipulate slice position.

Other siting considerations bear mention. Because iCMR remains investigational, the lab should be configured with ample space for multiple penetration panels near the control console, alongside the in-lab monitoring and display equipment. This enables new devices and control elements to be introduced without disturbing the radiofrequency shield. Multiple waveguides should be anticipated near the patient monitoring equipment, for non-conductive patient care equipment such as fiberoptic transmission lines. Other waveguides might be installed to facilitate LCD projectors outside the RF cage (see above). Inside the radiofrequency cage, multiple outlets should be installed for anesthesia gases on both sides of the magnet bore, including at a minimum medical air, oxygen, suction, and anesthesia exhaust. Multiple shielded AC electrical outlets should be installed, including connection to "emergency clinical power." There should be at least one sink inside the lab to dispose of biological fluids. Conceivably this sink can be prepared for surgical scrubbing.

Because equipment from the adjoining X-ray lab can be rolled into the MR lab, special training and operating procedures need to be established. Dual-use procedure tables and "Mayo-stands" should be MR safe. All equipment in the entire suite should clearly be labeled "MR-safe" or "MR-unsafe." "Unsafe" equipment that must be used in or near the MR system should be hard-tethered to the floors and walls to keep them away from the high fields (we use plastic chain-link tethers). Some laboratories draw magnetic field lines on the floor. To avoid inadvertent projectiles from staff pockets, some laboratories require pocket-less surgical scrub costumes.

Laboratories with double-door "RF lock" systems permit staff to enter and exit the scanner room without interrupting the RF shield. Other laboratories, including ours, tolerate the transient RF noise generated during real-time scanning as doors open and close.

MR gradients induce sufficient acoustic noise to interfere with voice communications, even using newer shielded gradient systems. Commercial systems are available that suppress or subtract acoustic noise; permit "open-microphone" communications among sterile staff without hands available for "push-to-talk" controls; and include multiple channels for parallel staff-patient and staff-staff communications. These may have pneumatic or fiberoptic transmission lines and, in the future, may have wireless capability. Washable wall and ceiling tiles with acoustic damping properties enhance hygiene and suppress acoustic noise.

#### Do animals belong in human iCMR suites?

iCMR systems require *in vivo *testing during investigational development, which requires either animal or human subjects. We believe rehearsal procedures and experiments in animals significantly enhance safety in patients. Many medical centers restrict or prohibit animal experimentation on clinical equipment largely on aesthetic or public relations grounds. We assert that human pathogens and blood products pose an immediate hazard of patient-to-patient infection yet animal tissue and dander are commonly ingested and pose little appreciable risk. The risk of allergic sensitization is mitigated by aggressive "operating-room" cleaning procedures after every animal experiment (and after every human procedure). In our dual-use facility, we have conducted hundreds of animal and human invasive and interventional procedures over seven years with no evident human infective or allergic complications.

Our MR and X-ray rooms are each equipped with separate air handling systems. All input air may be filtered (high efficiency particulate air, HEPA), all output air is exhausted from the building without recirculation ("single-pass air"), and the suite has a slight (negative) pressure difference compared with neighboring surgical suites, to contain animal odors.

Procedures for animal transportation were negotiated among interested parties in the hospital. Large mammals enter the building only under general anesthesia; are transported within the hospital on gurneys under blankets to disguise the contents; and are transported only along designated pathways and elevators to minimize contact with patients and lay visitors. All disposable equipment and medications intended for animal use are sequestered in a locked storage area during human procedures.

Separate equipment such as mechanical ventilators, defibrillators, intravenous pumps, and transport cradles are dedicated for animal use. Equipment connected by cable, such as hemodynamic recording, intravascular ultrasound, are permitted for dual use. Disposable plastic bags isolate equipment such as MR surface coils when used for animal experiments.

### Conspicuous and safe catheter devices

iCMR would probably already be in widespread use if conspicuous and safe clinical-grade catheters were readily available. The gap reflects the complexity of such devices and limited perceived return on industry investment.

Interventional catheter devices for X-ray procedures generally incorporate ferrous materials to impart mechanical characteristics (such as ability to transmit torque, to navigate tortuous structures, and to support insertion of less-flexible devices such as stents, endografts, and closure devices). Some, like widely-employed 316L stainless steel, are not significantly attracted by magnetic fields, but all create large imaging artifacts. As a result, most commercial braided diagnostic and guiding catheters, balloon-expandable stents, guidewires, and device delivery systems are not suitable for MR. Replacing the ferrous materials with "MR safe" materials such as polyester braiding usually renders the devices MR-inconspicuous. Other metals are better suited, including nitinol, many nickel-cobalt-chromium alloys (MP35N, L605, *etc*.), copper, gold, tungsten, titanium, and platinum, in that they create comparably minor magnetic susceptibility artifacts. Not all of these materials are biocompatible.

Conventional catheter components have intrinsic imaging "signatures" based on their X-ray attenuation that indicate catheter or guidewire tips, balloon inflation borders, transition zones where devices become stiff, secondary angulations where counterforce is applied, stent mount points, endograft sleeve components, docking locations, fixation pins, *etc*. These component imaging signatures are important to guide operators during interventional procedures, and replicating them under MR is a challenge. In case of device malfunction, even the distal shaft of a device may need to be visualized. Therefore the entire length of catheter devices must generally be visible for invasive procedures, not just the distal tip.

**Figure 3:**
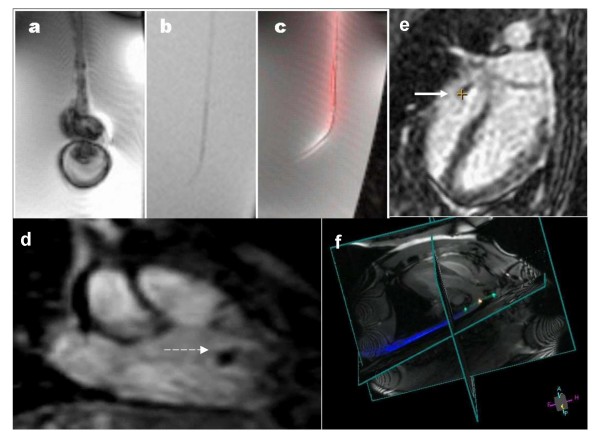
**MRI catheter designs**. (A) Conventional X-ray catheters are maneuverable in part because they contain steel braiding that distorts MR images. (B) With steel braiding removed, the same "passive" catheter is inconspicuous. (C) An "active" version of the same catheter in (B) has a loopless-design MRI receiver coil along its length, and another loopless-design guidewire receiver coil inside. Both are conspicuous along their shaft or profile. (D) A clinical grade balloon-tipped catheter can be filled with CO2 gas and appears as a black spot (dashed white arrow). This catheter has been manipulated from the femoral vein to the right ventricle in a patient (courtesy Reza Razavi, King's College London). (E) "Active tracking" in a porcine right ventricle (courtesy Steffen Weiss, Phillips Research Europe). A computer-synthesized cross-hair indicates the catheter position on an interleaved image. (F) Multiple channel "active imaging" catheter prototype where the shaft is seen in blue, proximal and distal tip microcoil markers are seen in green, and the catheter bend is seen as an orange signal.

Interventional MR catheter devices (Figure 3) are typically classified as "passive" or "active" depending on whether they are visualized based on intrinsic materials properties (passive) or based on signals from embedded receiver coils and electronics (active). Hybrids and combinations are also attractive options. The different approaches are summarized in Table 1.

**Table 1:** A comparison of catheter designs for interventional MRI.

**Approach**	**Advantages**	**Disadvantages**	**Examples**
Passive catheters, visible based on intrinsic materials properties	Simple, inexpensive Can be used in combination with other approaches	Non-specific catheter "signatures" "Compatible" conductive wires can heat Must not contain ferrous braids	Gadolinium-filled balloon dilatation catheters. Non-braided angiography catheters Polymer guidewires [57]
Active "imaging" catheters, incorporating MRI antennae	Highly conspicuous Catheters can be depicted in color Versatile imaging approaches including projection-mode	Complex, expensive Conductive wires can heat Blurry profile compared with X-ray catheters Dipole designs have poor distal tip visibility	Surgi-Vision *Intercept *0.030" dipole guidewire coil Boston Scientific *MRI Stilletto *endomyocardial injection system
Active "tracking" catheters, incorporating MRI antennae	Simple, inexpensive Coils can be tracked without imaging, to increase speed, reduce heating, reduce acoustic noise Coils can be used to automate scan plane adjustments	No real-time MRI Catheter locations are computer-synthesized on image Conductive wires can heat	MGH/General Electric electroanatomic mapping and MRI system [36]
Active catheters inducing susceptibility artifacts	Simple Controlled artifacts confer specificity	No imaging Heating of transmission lines when employed	Fiberoptic-detuned tracking coil [72]
Inductively-coupled devices	Requires no physical connection Reverse polarization mode may display unique device signature and allow high flip angle	Embeds electronics that might interfere with mechanical performance (*i.e*. on stents) Low flip angle compromises imaging	Essen wireless stents [69] and catheters [70]
Multispectral devices	Non-proton MRI species can be displayed in different colors from target tissue Passive imaging that does not require transmission lines or embedded electronics	Additional hardware required for exciting and detecting the alternate compounds Hyperpolarized ^13^C requires constant replenishment using specialized generator hardware	^13^C selective coronary arteriography [78] ^19^F catheter tracking [76]
Magnetically-deflected devices	Harness magnetic field to steer or even un-tether device	May compromise imaging May compromise mechanical capabilities	Untethered device navigation [75]
Integrated catheter MRI	Requires no conventional MRI Operates inside ordinary X-ray lab	Non-imaging device	Topspin intracoronary catheter [80].

As mentioned above, because most iCMR procedures use two-dimensional thin slices, parts of a flexible catheter may move outside the selected imaging slice. This is especially important with regards to the tip of a catheter. In this way iCMR and two-dimensional ultrasound-guided procedures differ substantially from projection X-ray fluoroscopy-guided procedures. In iCMR we compensate using interactive projection modes.

#### Passive catheters

Passive catheters are attractive in their simplicity but suffer from low contrast-to-noise ratios and non-specificity of image appearance. Passive devices are visible because of their intrinsic material properties. These may cause negative contrast through local distortions of the magnetic field (black spots) or positive contrast through enhanced local signal (bright spots). Black spots in an image can reflect many causes, and those created by passive devices (signal voids from intentional magnetic susceptibility artifacts) have low contrast compared with tissue. Black spots also suffer partial volume averaging artifacts more readily than do sources of intense signal (such as local receiver coils). Non-specificity refers to the inability to distinguish unique catheter image "signatures" from imaging artifacts. Vascular MR images have many long black lines that can be confused with black lines created by in-plane imaging of (susceptibility-based) passive catheters. This is not acceptable during catheter navigation near vital structures for interventional procedures. Susceptibility-induced black spots usually are much larger than the markers they represent. This "blooming" enhances visibility at the expense of extinguishing signal from surrounding target tissue.

Some groups use large devices, such as CO_2_-filled balloons [21], to displace water in order to make catheters conspicuous, to exploit their bloodstream flotation, and to minimize blooming. Others fill balloons with T2*-shortening agents to make them conspicuous and to distinguish them from blood during bright-blood imaging [22].

Several teams have developed off-resonance imaging sequences intended to make susceptibility marker catheters appear bright [23-25]. These can be safe and effective, but detract from tissue imaging and may depend on marker orientation with respect to the static magnetic field.

Unal and colleagues [26] and others have reported passive catheters coated or filled [27] with T1-shortening hydrogels or contrast agents, but follow-up reports have been limited. Technical challenges include accessibility of excited water spins to the contrast agent, enhanced T2*-effects of solid-phase or surface-affixed contrast agents, leaching, and replacing usable catheter volume (that otherwise could deliver large interventional devices) with contrast agents.

Given these limitations, we find the use of passive catheter devices alone to be generally inferior for interventional procedures.

#### Active catheter devices

Active devices have electronic components incorporated into their mechanical design. "Active-tracking" catheters incorporate receiver coils that are used with brief non-imaging pulse sequences to determine their position in space [28-35]. A common implementation is to interleave brief tracking pulses sequences with imaging pulse sequences, compute the coil coordinates, and to display position markers on the images to represent coil position. Alternatively, the position of active-tracking markers can be superimposed on prior or intermittently updated MR images [36].

One early active-tracking design induced local field inhomogeneities using direct current at the catheter tip [37,38]. This uses a blinking mode to impart specificity but suffers the disadvantages of both active and passive designs. A more recent design opposes solenoids for tracking as well as for high-sensitivity trans-axial imaging [39]. In addition to imaging and tracking, the device mounted coils can be used to estimate flow in real-time [40].

Robin Medical is commercializing a magnetometer approach for catheter tracking [41]. Position is determined based on timing of detected magnetic field gradients during imaging. Clinical products are available for neurosurgery, and investigational products are under development for cardiac electrophysiology MR catheters [41].

"Active-imaging" (also called "active-profiling") catheters incorporate receiver coils that contribute to the creation of MR images, and they are tracked based on their position within images. The catheter coils can be attached to separate receiver channels for reconstruction. Colorized display of catheter channels is a useful way to impart specificity to catheter-related signal [15,42,43]. Typically, active-imaging catheters are displayed with nebulous borders, unlike image depiction of X-ray catheters with sharp borders.

Simple active-imaging guidewires with a "loopless" design and 0.030" diameter (Surgi-Vision) have been tested clinically for catheter tracking or invasive imaging [44-46], and have even been reduced to 0.014" diameter [47]. These suffer limited antenna sensitivity near the tip, which reduces their clinical utility for navigating complex lesions and structures. More complex active-imaging guidewires with enhanced mechanical performance, shaft, and tip visibility, are under clinical development.

Active catheter devices usually operate in receive-only mode, but transmission-mode can dramatically reduce the local power absorption if it is not necessary to view surface coil images [48,49].

**Figure 4:**
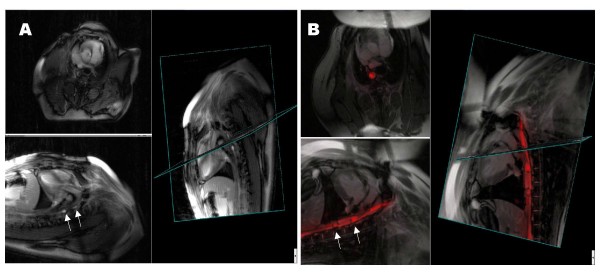
**A comparison of passive (A) and active (B) catheter visualization techniques during rtMRI stent angioplasty in a porcine model of aortic coarctation**. Panel A uses a passive PTFE-coated 0.035" nitinol guidewire, while panel B uses a loopless active 0.030" guidewire. The platinum-iridium (*Cheatham-Platinum*, Numed, Inc) stent is crimped on a dilute gadolinium-filled balloon (white arrows). The devices are more conspicuous and the procedures more straightforward using the active guidewire technique [51].

Imaging-active devices appear even brighter when used with contrast agents inside or alongside them [50], especially when combined with colorized display. For example, angioplasty balloons filled with dilute gadolinium contrast used together with active guidewires appear highly conspicuous and, by virtue of color-highlighted display, specific [51] (Figure 4).

#### Transmission lines, heating, and solutions

Conductive wires can resonate and heat during radiofrequency excitation for MR [52-54], whether part of active or passive devices. This has proven an important obstacle in implementing active catheter systems and in engineering mechanically effective catheters and guidewires. Very short wires are generally safe; very long wires can be safe also [55]. Resonating transmission lines also can deliver electric shock to the patient, especially when contacting myocardium, unless adequately insulated.

Fiberglass guidewires, doped with dysprosium susceptibility markers, were developed more than a decade ago [56] to avoid inductive heating, but had inadequate mechanical performance for human use. A nonconductive clinical-grade guidewire has been engineered by Bilecen's group in Basel [57] out of polyetheretherketone (PEEK) with improved mechanical performance. An iron-doped coating creates controlled susceptibility artifacts at the tip. The Philips Hamburg group describes a manufacturing technique called "micro-pultrusion" to create a non-metallic guidewire that compares favorably with widely-used clinical nitinol (*Glidewire*, Terumo) guidewires [58]. This wire has a short nitinol tip for performance and is doped or coated with iron, barium, and tungsten for visibility under MR and under X-ray. Because guidewires are fundamental tools for catheter procedures, these represent important enabling technologies for clinical interventional MR procedures in spite of the limitations of passive device tracking.

Heating has been attenuated in conductive transmission lines using coaxial chokes [59] and circuitry to detune or decouple during RF transmission[60], and by interposing serial transformers [61,62].

Atalar argues that any device can be safe from heating, as long as the radiofrequency excitation energy is limited appropriately [63]. Specific absorption rate (SAR) of radiofrequency energy is related roughly to the square of the static magnetic field strength, the square of the excitation flip angle, the square of the distance of conductive device from the center of the body, and inversely related to repetition time and to radiofrequency pulse duration. In terms of heating, this implies that for a given MR system, flip angle and peripheral location of conductive wires are "expensive" parameters. The target tissue also influences heat dissipation: flowing blood dissipates heat more than, for example, vascular occlusions.

Yeung and McVeigh [55] provide other important recommendations on techniques to limit heating of conductive devices. They assert that heating depends heavily on geometric parameters such as insertion length and depth of the catheter inside the body and with respect to the magnet isocenter and bore centerline. Very short and very long wires are desirable; very short and very long insertion lengths are undesirable. Eccentric wire position is undesirable as well.

Conductive transmission lines, for example using loopless (dipole-antenna) guidewires, have been considered more challenging for higher field MR. Paul Bottomley's lab recently reported loopless devices with decoupling and detuning circuitry at 3 T [64]. Despite long guidewires, both SNR and local sensitivity increased quadratically; as anticipated by Yeung and McVeigh, heating did not.

Other groups have replaced conductive transmission lines with fiberoptic cables, in one iteration using laser input to detune resonant circuits (creating controlled local dephasing "black spots") [65] rather than transmission of locally detected MR signals. Fandry and colleagues in Hamburg reported a fiberoptic transmission system that powers an active intravascular device[66]. It reduces SNR compared with electrical transmission lines but affords positive contrast and is an important advance. Michael Bock's group described an innovative optical gradient sensor that exploits the Faraday effect for catheter tracking, and uses an optical transmission line [67].

#### Inductively-coupled devices

Inductive tuning and coupling of catheter devices avoids heating of transmission lines and freedom from tethering altogether. Inductively-coupled intravascular devices amplify the local flip angle and the signal from adjacent tissues. These have been prototyped as intravascular stents which serve as the antenna [68,69]. Such devices can enhance internal signal, such as neointima or flow inside a stent or thrombus inside a cava filter. Unfortunately, bulky electronics may interfere with stent biocompatibility and performance. Inductive coupling has been used to implement safe "wireless" guiding catheters since the wire loops are short [70]. Practically, inductively-coupled devices require low flip angle imaging, which comes at the expense of surrounding tissue context, and may also require special miniaturized circuit elements to prevent heating.

**Figure 5:**
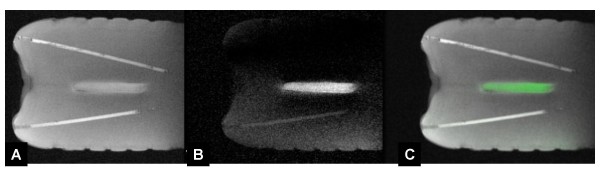
**A promising technique (reverse polarization) to impart "signatures" to inductively-coupled intravascular devices**. This is tested in a phantom using phased-array surface coils and real-time MRI. (A) Forward polarized mode shows the catheter and background. (B) Reverse polarization method distinguishes the inductively coupled receiver from background. The signal can be colorized and overlaid (C) as if it were an active catheter using a separate receiver chain. Courtesy Ergin Atalar, Bilkent University.

Without a transmission line, signal enhancement from inductively-coupled devices usually cannot be distinguished (for example, displayed in different colors) from signal enhancement from other surface coils. Duerk's group [71] used optical-detuning of inductively-coupled microcoils that were mounted on a catheter containing a fiberoptic transmission line. The resonant microcoils could be made to blink to aid in their localization and tracking. The Philips Hamburg group [72] implemented similar devices and methods to discriminate their signal from surrounding tissue. Atalar's group [73] recently described a method to detect "reverse polarized" local signal from inductively-coupled coils, and to distinguish it from surrounding anatomic signals. This innovation promises to provide safety advantages of passive devices, some of the conspicuity of active devices, and avoids the flip angle limitation of inductively-coupled coils (Figure 5).

#### Magnetic deflection and remote control

Magnetic deflection of catheter devices is commercially deployed outside of MR to aid in catheter steering and positioning. A University of Toronto team has reported electromagnet-deflectable catheters for interventional MR [74]. Martel and colleagues [75] have developed technically impressive integrated systems to navigate untethered devices within blood vessels during MR using customized gradient pulses. However, therapeutic devices using such "untethered" designs remain to be demonstrated.

#### Multispectral and other catheter designs

Another noteworthy approach is passive tracking of non-proton magnetic resonance species, which can therefore be distinguished from proton MR of surrounding tissue. The Kings College London team has reported tracking of catheters filled with 19-Fluorine [76]. The Amersham team uses catheters infused with hyperpolarized 13-Carbon [77] which also can be injected for selective angiography with high SNR [78]. These solutions combine the safety and simplicity of passive designs with the specificity of active designs to distinguish catheter components from target tissues.

One novel catheter zeugmatograph bears mention. The Topspin catheter is a self-contained catheter MR system that provides local intravascular diffusion-weighted measurements of coronary artery segments [79,80]. The system does not require a conventional MR system but does require intravascular balloon inflation to appose it to the artery wall because of its narrow field of view.

## Applications

### Applications suitable and not suitable for iCMR, including coronary intervention

Because MR affords flexible contrast and views at the expense of reduced temporal resolution or signal-noise, certain procedures are well suited for ICMR. These include tissues that are large or thick-walled relative to attainable fields of view (such as heart and aorta), tissue targets imaged with high contrast compared with surrounding tissue (such as myocardium against blood), and targets that are relatively immobile (such as peripheral arteries). Structures that can be contained within a single imaging plane (such as straight segments of iliac arteries) can be imaged rapidly, compared with small tortuous structures such as coronary arteries, which are difficult to image in real-time. Ventricular myocardium is especially attractive in spite of cardiac and respiratory motion because it is large, thick-walled, and well depicted with high contrast using SSFP compared with cavitary blood. Conversely, thin-walled cardiac atria are difficult to image even using segmented MR.

Speuntrup *et al *[81] delivered passively-visualized stainless steel stents in coronary arteries of normal swine. The devices and target proximal coronary arteries were imaged superbly using SSFP at 1.5 T despite cardiac motion and small targets. Unfortunately, barring significant technical breakthroughs, meaningful human coronary interventional procedures are unlikely to be performed under iCMR guidance. X-ray fluoroscopy offers high temporal (33–66 ms) and spatial (200–500 μm) resolution, allowing operators to navigate sophisticated 0.35 mm (0.014") diameter guidewires through small, heterogeneous, tortuous, calicified, and even occluded coronary arteries.

### Simple angioplasty and stenting procedures

Simple peripheral angioplasty is conducted effectively under X-ray guidance, so iCMR guidance is not compelling as a standalone application. Widely available, iCMR might be an attractive radiation-free alternative that also adds the ability to image uncommon complications such as peri-vascular hemorrhage.

MR has been used to conduct transluminal angioplasty [31,82-85] and stenting [51,69,86-92] in animal models of iliac, renal, and carotid arteries.

The team at the University of Regensberg has performed MR guided angioplasty and stenting in humans, using only passive devices, of iliac [93] and of femoropopliteal [94] atherosclerotic lesions.

### Aortic coarctation, aneurysm, and dissection

The aorta is the largest artery, and proximal aortic interventions are complex under any circumstances. X-ray alone is imperfect in visualizing complex three-dimensional aneurysms. Endovascular repair for aortic aneurysm is limited in part by endoleak due to inflow or outflow malapposition. Raman and colleagues [95] used MR to guide simple endograft tube repair of abdominal aortic aneurysm in pig models. Homemade temporarily-active endograft devices, which were depicted with color-highlighting until they were disconnected as RF coils after deployment, were more straightforward to use and more successful than comparable passive endograft devices. Endograft treatment under MR restored a normal lumen contour and laminar flow. Moreover, MR demonstrated device apposition to the target aortic wall and allowed interrogation for endoleak.

**Figure 6:**
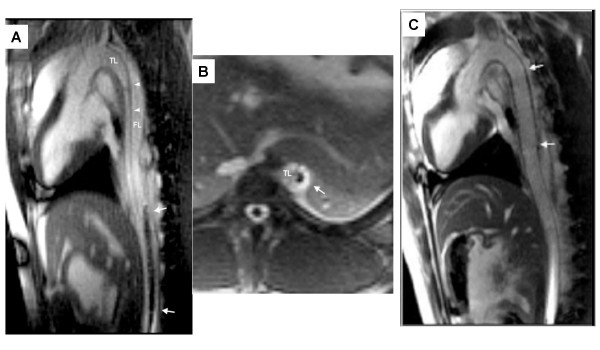
**Real time MRI guided endograft treatment of aortic dissection in swine**. The dissection flap (arrowheads) separates true lumen (TL) and false lumen (FL). (A & B) The passive endograft (arrows) has entered the false lumen and thereafter is redirected. Following endograft deployment, the false lumen is obliterated (Panel C). Courtesy Holger Eggebrecht, University Essen [96].

Eggebrecht and colleagues in Essen, Germany, elegantly applied iCMR to guide the placement of stent grafts in an animal model of thoracic aortic dissection [96]. They used off-the-shelf passive clinical self-expanding Gore *TAG *endografts without a guidewire. MR clearly revealed the true and false lumens of the dissected aorta, guided stent-graft deployment, and demonstrated stent-graft obliteration of the dissection and false lumen. This was an excellent demonstration of the value of simultaneous tissue and device imaging to guide a complex procedure (Figure 6).

Our team used active guidewires to treat an animal model of aortic coarctation with platinum-iridium stents [51]. Passive balloons filled with dilute gadolinium appeared bright over this active guidewire, and MR was helpful in displaying both target tissue and stent system during deployment. Velocity-encoded MR confirmed the hemodynamic results of the procedure. More important, MR provided immediate evidence of life-threatening rupture after intentional overstretch of the coarctation. In patients this recognition would provide precious additional time for rescue. Of note, the same procedure was far less effective when conducted using passive instead of active guidewires (Figure 4). Other teams [97-99] have modeled passive aortic endograft deployment in vitro or in vivo.

**Figure 7:**
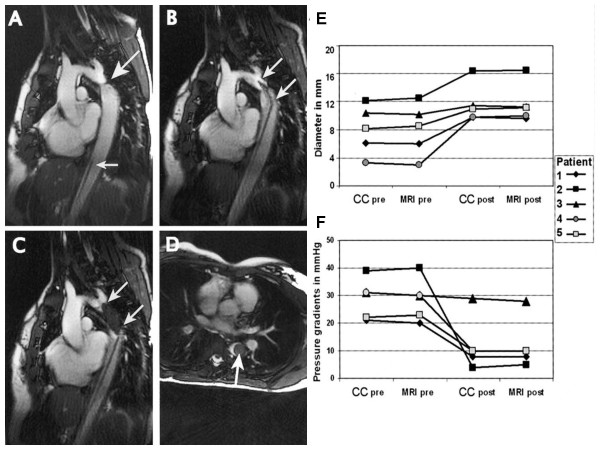
**Clinical demonstration of iCMR guided therapy of aortic coarctation**. (A) The large arrow points to the coarctation lesion. (B) A balloon dilatation catheter (double arrows) is positioned across the narrowing. (C and D) The balloon is inflated with a T2*-shortening agent. Panels E (aortic diameter) and F (pressure gradient) show favorable results of iCMR guided balloon angioplasty in 4 out of 5 patients (pre/post indicates measured before/after intervention). Courtesy Titus Kuehne, Charité Hospital [22].

Krueger, Kuehne, and colleagues in Berlin performed balloon angioplasty of aortic coarctation lesions in patients during MR. This was an important first clinical step toward wholly MR-guided treatment of this congenital lesion [22] (Figure 7).

### Chronic total occlusion

Perhaps more compelling are applications that exploit the ability of MR to visualize vascular spaces not conspicuous using X-ray. X-Ray with radiocontrast identifies the arteries up- and down-stream of the occlusion, but the occluded segment itself has no lumen to fill with radiocontrast and therefore remains invisible to the operator. "Blind" device manipulation risks procedural failure and arterial perforation. MR can visualize both patent and occluded artery segments, as well as the wall, lumen content, and adjacent structures, so it is attractive to guide recanalization [100].

Raval and colleagues created an animal model of peripheral artery chronic total occlusion in swine. They recanalized these lesions using homemade active devices under MR, even though they were fairly unsuccessful using X-ray and high-performance clinical guidewires [101]. MR helped operators traverse complex occlusions while keeping equipment inside the walls of the target artery. This is especially important in recanalization of tortuous peripheral artery occlusions, such as the iliac arteries.

Clinical-grade devices are currently under development to translate this experience into humans [102]. The group in Sunnybrook Hospital has developed a novel forward-looking catheter coil to guide recanalization using orthogonal solenoids.

### Valve Interventions and MR-Guided Cardiac Surgery

Non-surgical replacement of heart valves has become feasible, despite challenges in miniaturization, access, and image-guided positioning. MR might aid in positioning relative to vital structures such as coronary artery ostia. Kuehne and colleagues [103] have reported preliminary experience deploying a passively-visualized nitinol-based aortic valve prosthesis from a transfemoral approach in healthy swine. Their work demonstrates the value of combined device and tissue imaging for precise placement of critical prostheses. Such work may have value in complex pulmonary valve implants in patients with prior surgery for congenital heart disease.

Horvath, Guttman, and McVeigh at NIH have pursued operative MR guidance for cardiac surgery [104]. Using a minimally invasive incision to access the apex of the beating left ventricle, they have implanted commercial bioprosthetic heart valves mounted on stent devices into the aortic valve position. MR aided in axial and rotational positioning of the valve prosthesis with relation to the aortic annulus and coronary artery ostia. The same team is enhancing an MR-compatible robot for surgical applications [105]. MR-trackable thoracoscopes have been described to combine MR-derived anatomic "context" with optical imaging and direct instrument access [106].

### Congenital and structural heart disease

Diagnostic catheterization in patients with congenital heart disease is more complex and challenging than in adults with acquired heart disease, and may not be guided by ordinary anatomic predictions. Hearts after multiple palliative interventions have additional morphological complexity.

Schalla and colleagues at University of California San Francisco simulated clinical-grade pediatric diagnostic catheterization in an animal model of atrial septal defect, using active-tracking catheter devices. They combined invasive hemodynamic measurements with velocity-encoded MR [107]. Others have reported less complex procedures in animals [108,109].

The pioneering clinical diagnostic MR catheterization at Kings College London remains the landmark in the field [110]. Since this first report, they have conducted hundreds of diagnostic catheterization procedures in children using passive devices and CO_2_-filled balloons in a combined X-ray and MR suite (Figure 3d). In addition, they routinely conduct interventional procedures overlaying MR-derived roadmaps with live X-ray (see X-ray Fused with MR or XFM, below). Patients undergoing MR-assisted catheterization suffered significantly less radiation exposure than those undergoing conventional X-ray procedures.

The Kings College team [111,112] as well as Kuehne and colleagues [113] at Berlin Charité Hospital have used MR catheterization to make critical vessel-specific determinations of pulmonary vascular flow and resistance in patients with congenital heart disease or pulmonary artery disease. These may overcome inherent limitations of oximetry-derived measurements.

At least three teams have described iCMR guided deployment of nitinol occluder devices in animal atrial septal defects [114-116]. This application may not be superior to conventional or newer ultrasound guidance. Ventricular septal defect suffers additional morphological and procedural complexity that may benefit from iCMR.

### Connecting Chambers and Vessels

An especially attractive application of iCMR is the intentional violation of ordinary tissue spaces. A simple step in this direction is to connect adjacent vascular structures during atrial septal puncture. Arepally and colleagues at Johns Hopkins University [117] first described MR guided puncture of the interatrial septum using an active Brockenbrough-style needle. Our lab [45] conducted similar transseptal puncture and balloon septostomy, with MR assessment of the intracardiac shunts created. MR has particular value in specific identification and location of the "dangerous" tip compared with two-dimensional ultrasound. We did similar atrial septal puncture using an ablative laser catheter with an active-imaging distal tip coil [118], which by virtue of its flexibility could be applied in the future to more challenging geometry and more elaborate procedures.

Arepally and colleagues also used a similar active needle to effect a transcatheter mesocaval shunt, connecting the vena cava and the superior mesenteric vein using a nitinol connector [117]. They have also used this approach to deliver iron-labeled pancreatic beta-cell microcapsules under MR guidance to treat a model of islet-cell deficiency diabetes mellitus [119].

Using a unique double-doughnut MR configuration containing an integrated flat-panel X-Ray fluoroscopy system, Kee and colleagues at Stanford conducted preclinical [120] and clinical [121] transjugular intrahepatic portosystemic shunt (TIPS) procedures, which connect the hepatic and portal venous circulations in portal hypertension syndromes. MR guidance reduced the number of unsuccessful punctures, which can be hazardous.

Used this way, iCMR might enable non-surgical connections between other non-adjacent vascular structures, for example connecting the subclavian and pulmonary arteries, or the aorta and femoral arteries. Catheter-based extra-anatomic bypass might be an attractive alternative to open-surgical bypass, and is under active development. MR should permit inspection of vascular and extravascular spaces, the trajectory of connecting devices, and instantaneous assessment of the adequacy of catheter-based anastomoses.

### Cell and drug delivery to the myocardium

Several labs [25,122-129] have used iCMR and active or passive catheters to deliver cells and other materials into specified targets in normal and infarcted animal hearts. As described above, thick myocardial walls and high contrast with blood during SSFP MR afford excellent image guidance, and targeting based on multiple factors including late gadolinium enhancement, wall motion abnormalities, thickness, or anatomic position. Labeling or admixing injectate with contrast agents helps to confirm successful delivery, to assess intramyocardial redistribution, to assure confluent treatment volumes, or to avoid inadvertent injection overlap. The long-term value of exogenous intracellular contrast for tracking cell fate is at best controversial [130,131]. Promising therapeutic cell preparations have not yet been identified at the time of this writing. Should precise targeting be valuable for a future cell preparation, iCMR could be a valuable delivery tool.

### Image-guided Electrophysiology Procedures

In this family of procedures, cardiac rhythm pathways are mapped and interrupted by focused energy delivery or surgery. In catheter approaches, plans or even maps are routinely constructed using intracardiac electrograms and/or adjunctive X-ray, ultrasound, or imported tomographs. Multipolar pacing and ablation catheters are then guided by combinations of these images and maps and even can be directed by external magnetic deflection. Ablation lesions are interactively assessed by local heating and the impact on local and regional electrograms and rhythms, but not generally by imaging. In a surgical approach, anatomic myocardial segments are insulated from others under direct visualization. iCMR might afford surgical-style anatomic imaging guidance to catheter procedures, in positioning and in depicting actual ablation lesions during procedures [132].

MR with or without late gadolinium enhancement might have clinical value in identifying early tissue response to radiofrequency ablations [133-135], ablation lesions and perhaps lines of continuity as an imaging correlate of functional electrophysiologic block in thin walled atrial tissue [132,136,137], and the complex substrate of ventricular tachycardia [138]. MR already is in widespread clinical use overlaid with live X-ray or electroanatomical maps [139-142].

**Figure 8:**
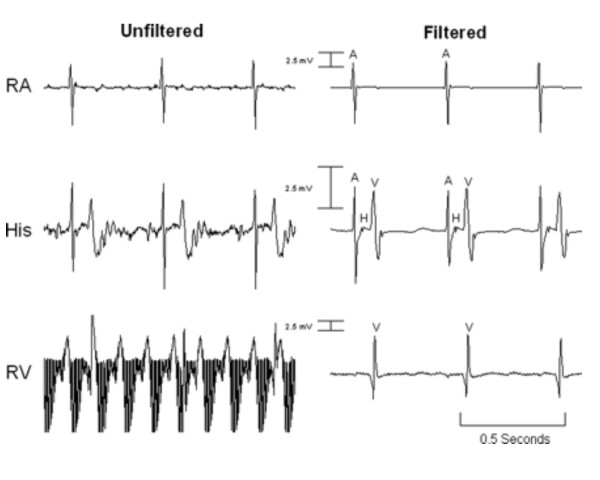
**Intracardiac electrograms are obtained during rtMRI in dogs without (left) and with (right) filtering using an MRI-compatible cardiac electrophysiology system**. The same system was used in two human patients. Courtesy of Henry R. Halperin, MD, Johns Hopkins University [144].

Henry Halperin's team at Johns Hopkins University first described active-imaging MR catheters with appropriate filtering to acquire intracardiac electrograms during real-time MR [143]. In a recent landmark study, they obtained first-in-man intracardiac electrograms in patients during MR [144] (Figure 8).

**Figure 9:**
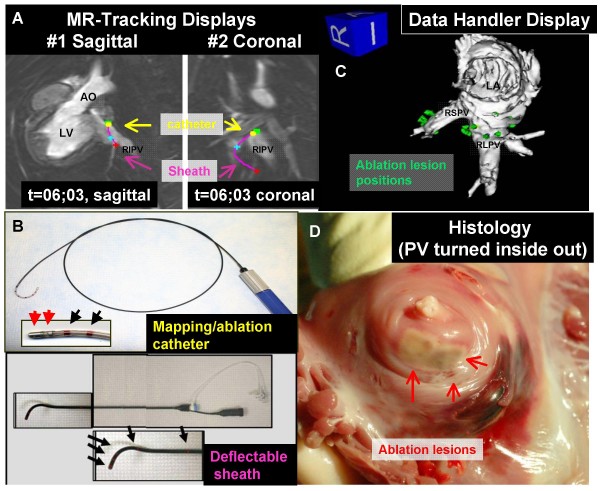
**Active-tracking microcoils are positioned in an MRI system using a prior time-resolved three-dimensional cardiac MR image**. Left atrium (around pulmonary veins) in a pig is evaluated using MRI compatible electrophysiology catheter and sheath system (Panels A & B). Ablation sites are mapped in an interactive three-dimensional display (Panel C) and seen on necropsy (Panel D). Courtesy of Ehud Schmidt, PhD, General Electric [36].

Vivek Reddy and colleagues at Massachusetts General Hospital in collaboration with General Electric [36] developed and tested novel real-time MR tracking of electrophysiology catheters in animals overlaid on high-resolution time-resolved MR images of myocardium obtained in the same system (Figure 9).

### Transarterial embolic protection, embolotherapy, and angiography

iCMR has been used to deploy prophylactic inferior vena cava filters in order to arrest ascending thromboembolism [145-148] and to visualize captured embolic material.

MR also has been used to guide visceral artery embolization, such as kidney segments, and affected parenchyma [149,150]. The Northwestern University team has coated polyvinyl alcohol particles with gadolinium chelates to monitor transcatheter arterial embolization procedures [151].

MR may have incremental value in tissue monitoring and assessment during and after interventional procedures, affecting for example renal [152] and myocardial parenchyma [153].

Clinically, Omary and colleagues at Northwestern University have been active in conducting clinical embolotherapy procedures in a combined X-ray and MR suite. MR perfusion assessment helped guide uterine artery embolization for leiomyoma (fibroids) [154]. Similarly they have used MR to monitor hepatic chemoembolization procedures for malignancy [155].

Bozlar and colleagues at University of Virginia [156] reported an innovative application of selective intraarterial contrast enhanced MR angiography. They subtracted intraarterial gadolinium MR contrast images from systemic (intravenous) contrast images to identify feeding collateral arteries in a pelvic arteriovenous malformation. Solely diagnostic applications of selective intraarterial contrast enhanced MR angiography [157] are less compelling than those combined with therapeutic procedures [16].

## Multi-modal image fusion: instant gratification

Multi-modal imaging systems are intended to provide some of the best features of both X-ray (simplicity and broad array of clinical catheter devices) and MR (soft-tissue landmarks).

### Hybrid X-ray MR Systems

Fahrig and colleagues at Stanford University developed a hybrid X-ray and MR system based on a double-donut MR scanner, with an X-ray source and detector mounted inside the magnet bore [158,159]. Images can be acquired alternately from X-ray and MR without moving the patient, providing images that are automatically co-registered. Several innovations have come out of the development of this system, such as X-ray transparent aluminum MR coils [160]. This system has been used clinically for transjugular intrahepatic portosystemic shunt (TIPS) creation [121] as described above. In addition, a system based on a 1.5 T closed bore system, with the X-ray gantry mounted just outside the magnet has been proposed [161].

### X-ray Fused with MR

MR roadmaps can be combined with live X-ray fluoroscopy using a conventional clinical environment and conventional catheter equipment, to provide additional anatomic landmarks and functional information to guide procedures [162,163].

In addition to the registration of the MR and X-ray coordinate systems, the X-ray system must be calibrated to compensate for image distortions. These orientation-dependent distortions are caused by C-arm imperfections (e.g. pincushion distortions, gantry fatigue) and the influence of the MR system's main magnetic field on the X-ray image intensifier. Using two-dimensional and three-dimensional phantoms with fiducial markers at known locations, warping polynomials may be calculated which can be used for distortion correction [164,165]. These distortions are less important using contemporary flat-panel X-ray detectors.

In order to merge MR and X-ray images, the coordinate systems of each must be aligned. The approach taken by Razavi is to use a calibration phantom, which can accommodate X-ray or MR compatible markers, to compute the transformation between MR scanner space and X-ray table space. The position of the X-ray components are tracked using infrared emitting diodes placed on the X-ray C-arm and table. An additional marker may be placed on the patient's chest to allow for adjustments to the registration to be made in case of gross patient motion [140,162].

In our lab, registration is accomplished by placing multi-modality external fiducial markers either directly on the patient's skin or in an elastic band around the patient's torso. The markers contain a mixture of iodinated X-ray contrast and gadolinium based MR contrast, so that they can easily be identified in both imaging modalities. A three-dimensional MR T1-weighted acquisition and a set of multiple baseline X-ray projections are used to register the two coordinate systems. Using custom built software, the three-dimensional positions of the markers are automatically reconstructed from the MR and X-ray images, correspondence between the markers is established, and a three-dimensional rigid registration algorithm is used to align the MR coordinate system with the X-ray coordinate system.

**Figure 10:**
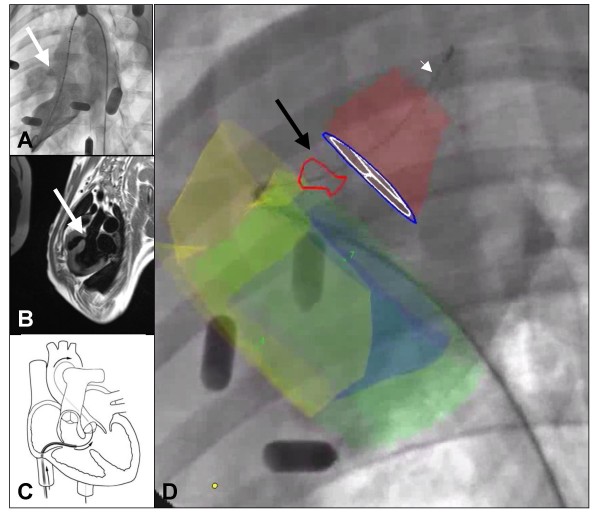
**X-ray Fused with MRI (XFM) guided repair of a membranous ventricular septal defect in swine **[167]. Panel A shows traditional X-ray contrast left ventriculogram highlighting ventricular septal defect (VSD; thick, white arrow). Panel B shows turbo spin-echo 3 chamber view, which highlights the VSD (thick, white arrow) in the same orientation as X-ray image in panel A. MRI regions of interest were segmented manually to be presented to the operator as a static image overlay on real-time X-ray fluoroscopic images. Panel C is a schematic depicting a new XFM guided antegrade approach to crossing the VSD in order to close the defect with a nitinol implant. XFM roadmap (Panel D) elements include the right ventricular endocardial contour (yellow), the left ventricular epicardial contour (green), the left ventricular endocardial contour (blue), the VSD tract (red outline and black arrow), aortic root (red), and aortic valve (blue annulus with white leaflets). The guidewire (white arrowhead) is delivered across the VSD and into the aorta.

Relevant anatomic landmarks (e.g. myocardial surfaces, the great vessels, cardiac valves) and treatment sites (e.g. ventricular septal defect, myocardial infarct, injection target) are segmented on MR images and can be color-coded. Two-dimensional projections of these three-dimensional contours are constructed based on the X-ray gantry orientation, registration calculations, and calibration data. These projections are overlaid on the X-ray fluoroscopy images and displayed in the angiography suite (Figure 10). Position information from the X-ray system (c-arm angle, table position, magnification factor) is used automatically to adjust the displayed MR derived contours with the fluoroscopic images during a procedure.

The Kings College London team uses X-ray Fused with MR (XFM) to provide soft tissue context to clinical electrophysiology procedures [162] and pediatric interventional procedures. Our lab has used XFM to guide cell delivery to infarct targets with millimeter precision, using targets such as infarct borders or zones of excessive thinning [166]. We also have used XFM to guide a novel antegrade approach to catheter-based repair of membranous ventricular defect in swine (Figure 10), which significantly shortened X-ray exposure compared with the conventional retrograde approach [167], as well as a novel approach to catheter-based mitral annuloplasty involving an intramyocardial guidewire trajectory. In patients we have used XFM to facilitate biopsy of high risk cardiac structures, and to aid in recanalization of chronic total peripheral artery occlusion [163].

A significant limitation of the XFM type roadmap approaches is that out-of-date roadmap information can be misleading or hazardous. Roadmap information can be distorted by rigid devices, by changes in the underlying tissue structure related to the intervention, and from uncorrected cardiac and respiratory motion.

## Conclusion

We have outlined how and why to configure an interventional CMR facility. The incremental cost of an intermodality transportation system and of barrier doors is relatively low. Existing technology already provides excellent real-time MR imaging sufficient to guide investigational therapeutic procedures, and to care for patients in this environment. Clinical grade catheter devices remain the last persistent obstacle to widespread investigational use of iCMR.

Important competing technology is available. Electromagnetic position mapping relies on prior (roadmap) anatomic information. CT provides superb soft-tissue imaging but even newer massive detector arrays expose patients to prohibitive doses of ionizing radiation to guide interventional cardiovascular procedures. C-arm based cone-beam CT techniques create effective soft-tissue roadmaps, but radiation exposure limits the ability to inspect the impact of procedures. Three-dimensional surface and transesophageal ultrasound provides some of the functionality promised by MR with little fuss, albeit with inferior acoustic windows.

MR may prove superior because it provides single-station imaging, combining three-dimensional anatomy, biochemical characterization, mechanical function, and hemodynamics. Real-time MR provides "surgical" exposure that can revolutionize minimally invasive procedures. The mythological figure Tantalus was punished with temptation by fruits and drink eternally out of reach. Readers of reviews like this have long been tempted by fruits of MR-guided intervention. Seemingly eternally tantalizing, clinical interventional CMR is nearing our grasp.

## Abbreviations

MR: Magnetic resonance imaging; iCMR: Interventional CMR; SSFP: Balanced steady state free precession pulse sequence

## Competing interests

NIH and Siemens Medical Systems have a collaborative research and development agreement for interventional CMR and multimodality image fusion. MAG, OK, and RJL are inventors on patents or patent applications, assigned to NIH, on MR techniques or devices with applications in iCMR. No other financial conflicts of interest are identified.

## Authors' contributions

KR and RJL drafted the initial review article, RJL performed the literature search, AZF, MAG, OK, and CES contributed article content and participated in editing and final drafting of the manuscript. All authors read and approved the final manuscript.
